# The Biologic IRL201805 Alters Immune Tolerance Leading to Prolonged Pharmacodynamics and Efficacy in Rheumatoid Arthritis Patients

**DOI:** 10.3390/ijms25084394

**Published:** 2024-04-16

**Authors:** Christopher Hall, Jill Pleasance, Oliver Hickman, Bruce Kirkham, Gabriel S. Panayi, Paul Eggleton, Valerie M. Corrigall

**Affiliations:** 1 Academic Department of Rheumatology, Centre for Inflammation Biology and Cancer Immunology (CIBCI), King’s College London Faculty of Life Sciences and Medicine, Guy’s Hospital Campus, London SE1 1UL, UK; 2 Revolo Biotherapeutics, London SE1 9AP, UK

**Keywords:** autoimmunity, biologic therapy, biomarkers, inflammation, regulatory T cells, cytokines

## Abstract

A homologue of binding immunoglobulin protein/BiP—IRL201805 alters the function of immune cells in pre-clinical in vivo and in vitro studies. The aim of the study was to select biomarkers that clearly delineate between RA patients who respond to IRL201805 and placebo patients and reveal the immunological mode of action of IRL201805 driving the extended pharmacodynamics observed in responding patients. Biomarkers that distinguished between responding patients and placebo patients included downregulation of serum interferon-γ and IL-1β; upregulation of anti-inflammatory mediators, serum soluble CTLA-4, and intracellular monocyte expression of IDO; and sustained increased CD39 expression on CD3^+^CD4^+^CD25^hi^ CD127^lo^ regulatory T cells. In the responding patients, selected biomarkers verified that the therapeutic effect could be continuous for at least 12 weeks post-infusion. In secondary co-culture, pre-infusion PBMCs cultured 1:1 with autologous PBMCs, isolated at later time-points during the trial, showed significantly inhibited IL-6 and IL-1β production upon anti-CD3/CD28 stimulation demonstrating IRL201805 alters the function of immune cells leading to prolonged pharmacodynamics confirmed by biomarker differences. IRL201805 may be the first of a new class of biologic drug providing long-term drug-free therapy in RA.

## 1. Introduction

Rheumatoid arthritis (RA) is a chronic inflammatory autoimmune disease predominantly affecting females (3:1) leading to debilitating pain and joint degeneration, with a prevalence of approximately 1% worldwide. The burden of increased morbidity and mortality associated with RA has driven the rapid development of therapeutics during the last three decades. From non-steroidal anti-inflammatory drugs (NSAID) and disease modifying anti-rheumatic drugs (DMARDs) to the biologic DMARDs (bDMARDs) including small-molecule inhibitors, these drugs now target the inhibition of pro-inflammatory mediators, such as cytokines, or cell signalling cascades [1].

Despite the fact that bDMARDs have greatly improved the quality of life for many patients, these drugs still tend to treat the symptoms rather than ‘cure’ the disease. One barrier to this goal of long-term drug-free remission remains the limited mechanism of action of the majority of currently available bDMARDs. Other considerations include the health risks associated with the development of serious infections or cancers or the poor efficacy of a drug in restricting a biological pathway [2]. The limiting factor for many of these drugs is the close association between the pharmacokinetics (PK) and pharmacodynamics (PD) requiring regular dosing with the drug. However, for prolonged drug-free efficacy, the bDMARDs must critically change the cellular immune response. There is a need for a drug that has the potential to deliver long-term alteration of cell surface phenotypes, production of pro- or anti-inflammatory soluble mediators and modify downstream signalling pathways, leading to a significantly prolonged PD beyond PK. Such a molecule is Binding immunoglobulin Protein/glucose regulated protein 78 (BiP/GRP78), a ubiquitous, endoplasmic reticulum resident protein essential for the regulation of protein folding. The intracellular functions of this protein are well characterised [3].

However, extracellular BiP/GRP78 is also defined as a stress protein, and exerts markedly different effects on the immune response, driving the production of anti-inflammatory mediators [4]. In the human immune system, the anti-inflammatory activity of extracellular BiP is principally manifested through myeloid cells that express an as-yet unidentified receptor(s) for BiP. BiP stimulation of monocytes diverts classical myeloid activation to alternative activation, or deactivation. BiP influences the differentiation of peripheral blood monocytes (PBMO) into dendritic cells (DC) and osteoclasts. Treatment of monocytes with BiP, while undergoing maturation to DC, reduced their expression of HLA-DR and downregulated CD86, diminishing their critical antigen presentation function. In animal studies, administration of a single dose of recombinant human BiP prophylactically protects and therapeutically treats animals with murine collagen-induced arthritis (CIA) [5,6]. Earlier, a single parenteral dose of BiP during active disease was sufficient to induce the remission of inflammation in CIA, suggesting that BiP not only mediates anti-inflammatory actions but also drives the resolution of inflammation [5].

This led to the initiation of our previously reported ‘first in human’ double-blind randomised placebo controlled single escalating dose phase I/IIa trial of the homologue of BiP/GRP78, now called IRL201805 [7] in RA (RAGULA trial). During this trial, clinical biomarkers were comprehensively reported. Those included were the disease activity score 28 joint count (DAS28), and serum inflammatory markers such as C-reactive protein (CRP) -interleukin (IL)-8 and the vascular endothelial factor (VEGF), which impact disease pathology and are regularly used as disease activity markers. However, the disconnect between PK and PD additionally required the creation of a panel of biomarkers that clearly identified significant immunological differences between the placebo (Pbo) group and all those patients treated with IRL201805, whether responsive (IRL201805Res) or non-responsive (IRL201805NRes), which would confirm that treatment with IRL201805 was responsible for the changed immune response, forcing the resolution of chronic inflammation in disease.

Herein, we describe changes in influential exploratory biomarkers that increase our understanding of the mechanism of action of IRL201805 that underlie the potential for resolving inflammation and restoring homeostasis in a drug -free environment. This study shows the effect of IRL201805 on the production of soluble mediators, with significant differences between placebo and IRL201805Res patients: in vitro and ex vivo cell surface phenotypic changes; altered regulatory T cells (Treg) markers; and finally, confirmation that altered immune cell function was established during treatment as demonstrated by the altered cytokine profile produced by IRL201805Res patients for 12 weeks post treatment.

## 2. Results

### 2.1. Disease Activity as Estimated using DAS28 Was Consistently Reduced in IRL201805 Responders

**Figure 1:**
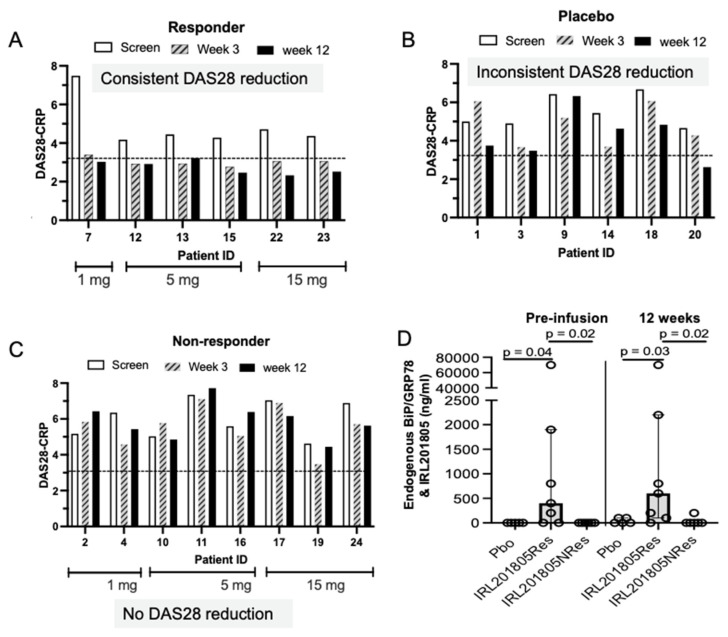
Clinical biomarkers and detection of serum BiP/GRP78 and homologue IRL201805. (**A**–**C**) Three different single doses of IRL201805 (1, 5, 15 mg/human) or placebo were infused randomly into patients entering the RAGULA trial. Disease activity scores from a 28 joint count (DAS28), using ESR, were monitored over the 12 weeks of the trial. Each patient’s score is shown for pre-infusion (screen) and at 3 weeks and 12 weeks post infusion. Dotted line represents the DAS28 score 3.2, representative of low disease activity cut-off. (**D**) Serum BiP/GRP and IRL201805 homologue concentration was monitored in sera using enzyme linked immunoassay from before administration until 12 weeks after the single intravenous infusion.

The DAS28 is a measure of disease activity in RA based on the clinical assessment of 28 specific joints, serum inflammatory markers (e.g., erythrocyte sedimentation rate—ESR or CRP) combined with a visual analogue scale. The DAS28 of the patients was monitored for 12 weeks (Figure 1A–C). The pre-assessment DAS28 was > 4 for all patients. Retrospectively, it was found that all Pbo and 7/8 non-responders had a DAS28 of ≥5. Using the EULAR definition [8], a reduction in the pre-infusion DAS28 score to 3.2 (low activity level) or 2.6 (remission level) when sustained for up to 12 weeks defined IRL201805Res (Figure S1). These responder patients showed a consistent low-level DAS28 from 3 to 12 weeks post-treatment (Figure 1A). The remainder of the IRL201805-treated patients showed inconsistent and transient changes in DAS28 and were grouped as IRL201805NRes (Figure 1C). Despite a detectable clinical response, the DAS28 in the Pbo patients showed only fluctuating, transient changes (Figure 1B). The IRL201805Res group had statistically significant lower DAS28 scores compared to either Pbo or IRL201805NRes patients from week 3 onwards (Figure S1). During the trial, samples were taken to investigate exploratory biomarkers; however, the small number of patients necessitated the pooling of data across the three dosing groups (1, 5, and 15 mg/patient) to form the IRL201805Res and IRL201805NRes groups.

### 2.2. Serum Endogenous BiP/GRP78 and IRL201805 Concentrations Were Higher in IRL201805 Responders

Throughout the RAGULA trial the serum level of endogenous BiP/GRP78 and the IRL201805 homologue, which are indistinguishable using ELISA, was measured. BiP/GRP78 is an endogenous protein and ELISA data showed that pre-infusion the RA patients had a range of serum concentrations (0–70 µg/mL). Despite the randomisation of patients for placebo or IRL201805 treatment, on unblinding at the trial end, these pre-infusion serum levels revealed that the IRL201805Res group had significantly higher endogenous levels of serum BiP/GRP78 prior to treatment, while the Pbo group and IRL201805NRes patients had little detectable endogenous serum BiP/GRP78 (Figure 1D). We followed the first 72 h post-infusion to investigate whether serum levels in individual patients changed from their pre-infusion level, however, no patient showed any serum concentration change for BiP/GRP78/IRL201805.

### 2.3. Pharmacokinetic–Pharmacodynamic (PKPD) Analysis of IRL2028 in Mice

**Figure 2:**
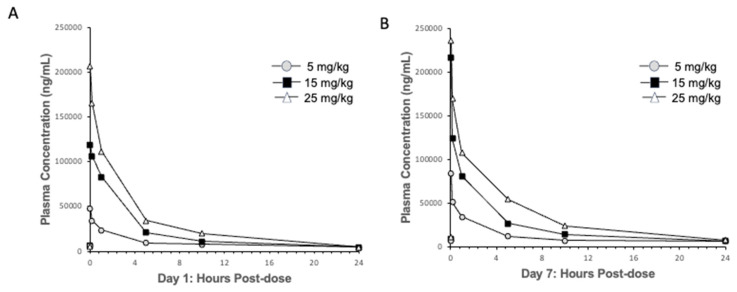
Pharmacokinetics of IRL201805 in mice. (**A**) The disappearance of IRL201805 from the sera of mice given three increasing doses from 5 to 25 mg/mouse, and (**B**) the time-course repeated one week post the original infusion.

The findings of the RAGULA clinical trial confirmed a complete disconnect between IRL201805 PK and PD, which was previously observed in a pre-clinical-collagen-induced arthritis model [5]. Regulatory restrictions prevented PK measurement in either healthy controls or RA patients, so the investigation of IRL201805 PK was limited to animal models. Monitoring the PK in the mouse showed a rapid loss of serum IRL201805 following infusion of each of the doses of IRL201805 with a t _1/2_ < 3 h and complete loss from the serum by 10–24 h (Figure 2A). With repeat dosing after 1 week, the PK followed the same kinetics (Figure 2B).

### 2.4. Detection of Cytokine Changes Post ILR201805 Treatment

As previously reported, a single-dose infusion of IRL201805 in RA patents led to the significant inhibition of inflammatory biomarkers C-reactive protein (CRP), IL-8 and vascular endothelial growth factor (VEGF) specifically in IRL201805Res patients when compared to the placebo (Pbo) group. These changes were already visible by 3 weeks and persisted until week 12 [7].

Interleukin (IL) IL-2, IL-4, IL-6, IL-10, IL-17, IL-1 receptor antagonist (Ra), tumour necrosis factor (TNF) α, interferon (IFN) γ, cytotoxic T lymphocyte antigen (CTLA)-4, and monocyte chemoattractant protein (MCP)-1. These were all detectable, but of these only IL-1β, TNFα, and IFNγ showed statistically significant changes in serum concentration between the groups, as noted (Table 1). As a means of predicting and monitoring drug treatment in rheumatoid arthritis, cytokines have been considered as potential biomarkers to use in parallel with CRP and ESR to monitor disease activity, but even these established acute phase-reactant levels can be discordant among RA patients with active disease [9]. IL-10 is an important anti-inflammatory in the rheumatoid joint and synovial tissue, where it binds to IL-10 receptors on a number of immune cells, including Tregs. However, the monitoring of free soluble IL-10 levels as a strategy to predict disease amelioration is not routinely used. Meyer and co-workers showed that serum IFNγ, IL-1β, IL-1R, TNF-α, GM-CSF, and VEGF significantly correlated with disease activity in patients with high DAS28 scores [10]. Moreover, the IRL201805 study has also shown a reduction in IFNγ, IL-1β, VEGF, and CRP in the clinically responding patients specifically [7]. Others have shown that serum levels of IL-6, IL-10, and IFNγ have modest correlation when assessed with the radiological progression of RA [11], but we did not perform this correlation in the current study. As shown in Figure S2, there was a wide range of IL-10 concentrations in all three patient test groups where the IRL201805Res group appeared to have generally higher levels of IL-10 compared to the other test groups throughout the study. We saw no correlation with IL-10 concentrations and drug dosage.

**Figure 3:**
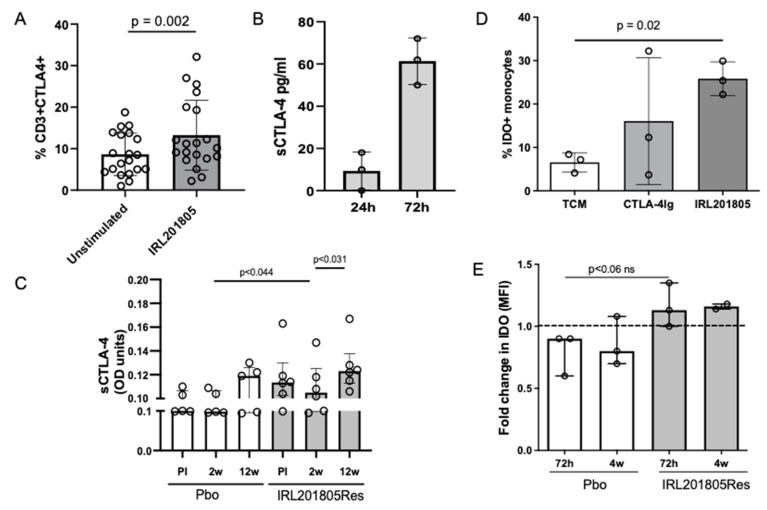
Cytotoxic T lymphocyte-4 is induced by IRL201805. (**A**) In vitro culture of peripheral blood mononuclear cells (PBMCs) from RA patients for 24 h with and without IRL201805 were stained and analysed using flow cytometry for expression of intracellular and cell surface CTLA-4 on T cell. n = 23. (**B**) Secretion of sCTLA-4 into tissue culture medium over time was monitored over 72 h. sCTLA-4 was measured using ELISA (n = 3). (**C**) sCTLA-4 detected in patient’s serum using ELISA from the placebo (Pbo) and IRL201805 responsive (IRL201805Res) patients. (**D**) The induction of intracellular indoleamine dioxygenase (IDO) in monocytic cells by CTLA-4Ig or IRL201805 after culture for 72 h. (**E**) Detection using intracellular flow cytometry comparing the frequency of cells expressing IDO in Pbo and IRL201805Res patients at different time-points as indicated. The fold increase from pre-infusion is shown (n = 3). Ns = non-significant.

In PBMC in vitro studies, we observed IRL201805 upregulated expression of the cell surface CTLA-4 on RA peripheral blood mononuclear cells (PBMCs) compared with healthy control samples (control, 8.6 ± 5.1% vs. IRL201805, 13.2 ± 8.4%, n = 23, *p* = 0.02) (Figure 3A). We explored these findings further as CTLA-4 derives from activated T-cells, especially Tregs, and can bind to CD80/CD86 on APCs with higher affinity than CD28. Abatacept, a CTLA-4-Ig fusion protein, binds to CD80/CD86 and blocks inflammatory pathways in T-cells and is used to treat progressive RA [12]. Mechanistically, evidence suggests soluble CTLA-4-Fc induces tolerogenic conditions via indolamine dioxygenase (IDO) and Tregs, by increasing the production of enzymatically active IDO, but not necessarily increasing the Treg number [13]. In physiologically relevant whole-blood cultures, the addition of IRL201805 induced soluble CTLA-4 (sCTLA-4) production (24 h, range 1–18 pg/mL; 72 h, range 50–72 pg/mL) (Figure 3B) when the control cultures showed no detectable CTLA-4. Monitoring RAGULA-trial derived sera, sCTLA-4 levels between pre-infusion and 12 weeks, both placebo and IRL201805Res groups showed a slight drop in serum sCTLA-4 at 2 weeks; although, sCTLA-4 levels in placebo patients remained significantly lower than the IRL201805Res group (*p* < 0.044) (Figure 3C). Within this group, significant upregulation of serum sCTLA-4 (*p* = 0.031) was detected by 12 weeks.

This posed the question as to whether IRL201805, like CTLA-4Ig, could induce IDO, a monocyte and DC anti-inflammatory mediator that metabolises tryptophan, thus inhibiting T cell proliferation. In following in vitro experiments, PBMCs were cultured with CTLA-4Ig [14] or IRL201805 for 24 h. A rapid increase in intracellular IDO was observed in myeloid cells after treatment with both CTLA4Ig and IRL201805. The increase in the IRL201805-treated cells vs. control (TCM) cells was statistically significant (*p* = 0.02) (Figure 3D). Similarly, ex vivo samples from IRL201805Res patients revealed a strong trend (*p* < 0.06) at 72 h for greater intracellular expression of IDO when compared with Pbo at the same time-point. This increased expression of IDO appeared to be maintained for 4 w (Figure 3E).

### 2.5. IRL201805 Is Not a General Immunosuppressive

**Figure 4:**
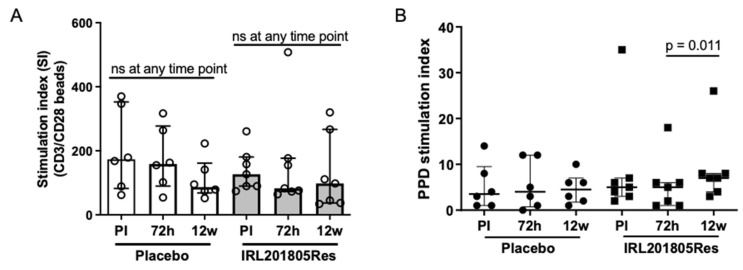
IRL201805 is not an immunosuppressive drug. RA peripheral blood mononuclear cells (PBMCs) samples from the placebo (Pbo) or responsive IRL201805-treated patients (IRL201805Res) pre-infusion, at 72 h or 12 w, were (**A**) cultured for 72h in the presence or absence of anti-CD3 and antiCD28 antibody coated beads, or (**B**) cultured for 120h in the presence or absence of tuberculin purified protein derivative (PPD). Proliferation was measured by addition of tritiated thymidine to cultures for the last 24 h. Ns = non significant.

The possibility that IRL201805 might be generally immunosuppressive in patients was excluded by a comparison of patient’s PBMC proliferative responses pre- and post- treatment. The data showed no reduction in T cell responses either to anti-CD3+anti-CD28 antibody coated beads or tuberculin-purified protein derivative (PPD) in any patient group at any time point throughout the 12 weeks of the trial (Figure 4A,B, respectively). It was noted, however, that the recall antigen cell-mediated immune response to tuberculin PPD was significantly raised (*p* = 0.011) in the IRL201805Res patients between 2 and 12 weeks. 

### 2.6. Frequency of Phenotype Changes of Regulatory T Cells

**Figure 5:**
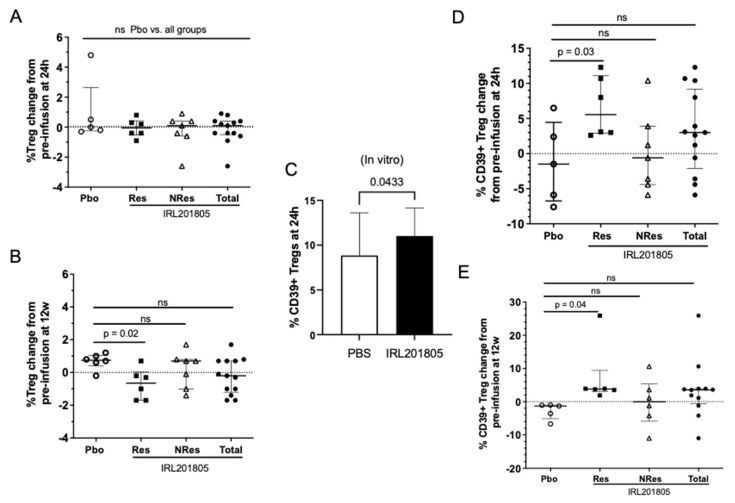
IRL201805 alters regulatory T cell phenotype. Ex vivo heparinised peripheral blood samples were stained for regulatory T cell phenotype CD45^+^CD3^+^CD4^+^CD25^hi^CD127^lo^ (Treg) in whole-blood samples and monitored over 12 weeks. (**A**) 24 h and (**B**) 12 w timepoints are shown (n = 6). (**C**) In vitro preincubation of RA peripheral blood mononuclear cells (PBMCs) in tissue culture medium with or without IRL201805 for 24 h before staining with anti-CD39 and anti-CD73 fluorochrome conjugated antibodies and analysis using flow cytometry (n = 23). (**D**,**E**) Whole-blood samples as in (**A**,**B**) stained for Treg phenotype described above and CD39 expression (n = 6/group). NS = non significant.

An established Treg phenotype, CD3^+^CD4^+^CD25^hi^CD127^lo^, was monitored ex vivo in patient samples over 12 weeks. Figure 5A,B show that at 12 weeks post-infusion a slight but significant fall in circulating Tregs was observed in IRL201805Res patients only when compared to Pbo patients (12 weeks, Pbo, % of T cell population, 6.6 ± 2.7%; change from baseline, 0.41 ± 4.8 vs. IRL201805Res, % of T cell population, 6.0 ± 2.0%; change from baseline, −0.76 ± 12.96; change in expression from baseline *p* = 0.017) (Figure 5A,B).

In a previous in vitro culture, the cell surface phenotype following 24 h in culture in the presence of IRL201805 showed significant upregulation of CD39 with CD73 on the surface of the CD4+CD25hi regulatory T cell subpopulation (Figure 5C). CD39 has become a marker for stability and the increased efficacy of the CD4^+^CD25^hi^CD127^lo^ Treg [15], so for consistency the frequency of expression of CD39^+^ was also monitored. The ectoenzyme CD39 was upregulated and was significantly higher on IRL201805Res Treg by 24h (Figure 5D) and maintained for at least 12 weeks post-infusion in the absence of further dosing (Pbo, % expression, 38.4 ± 19.9%, an increase from mean pre-infusion expression, 0%, vs. IRL201805Res, % expression, 54.0 ± 25.2%, an increase from mean pre-infusion expression, 6.6%, *p* = 0.017) (Figure 5D,E).

### 2.7. IRL201805Res Treatment Confers an Altered Cytokine Profile

To interrogate any functional changes that might have occurred, in vivo PBMCs from Pbo or IRL201805Res patients were taken at 72 h or 4-week time-points and cultured 1:1 with autologous pre-infusion PBMCs stimulated with anti-CD3+anti-CD28 antibody coated beads.

The proliferative response was extremely variable within the groups, and consequently no significant difference between the IRL201805Res group and Pbo group was observed.

**Figure 6:**
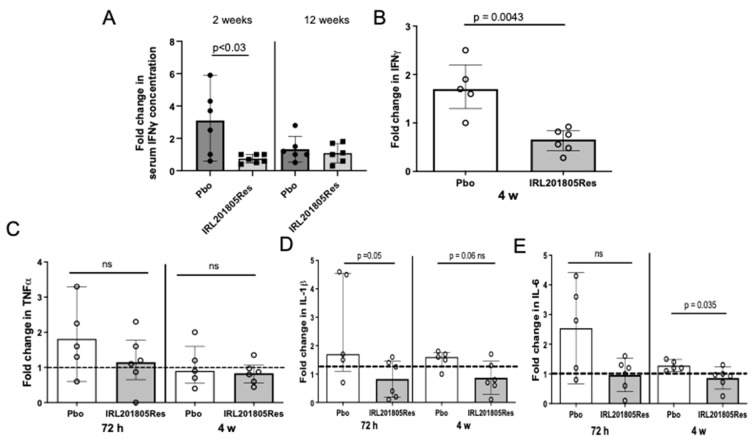
IRL201805 treatment changes inflammatory cytokine release by immune cells from responding patients. (**A**) Fold change from pre-infusion serum IFNγ levels in placebo (Pbo) vs. responding patients (IRL201805Res) at 2 weeks and 12 weeks. (**B**–**E**) Peripheral blood mononuclear cells from pre-infusion were cultured 1:1 with autologous PBMCs from later time-points, as stated. After 24 h, Luminex technology was used to measure (**B**) IFNγ, (**C**) TNFα, (**D**) IL-1β, and (**E**) IL-6 in the supernatants (n = 6). All data were recorded in replicate. Data shown as fold-change from pre-infusion cells alone. Ns = non significant.

Serum IFNγ levels were significantly reduced compared to pre-infusion levels from IRL201805Res patients at the 2-week timepoint compared with Pbo (*p* < 0.03) (Figure 6A). Taking PBMCs from these blood samples for co-culture with autologous, stimulated pre-infusion PBMCs the IRL201805Res 4-week samples also produced significantly less IFNγ than the equivalent Pbo cultures (Figure 6B). In these same cultures, TNFα production, although not significantly decreased (Figure 6C), showed a strong trend towards reduced production in the IRL201805Res cultures. These cultures also showed a significant reduction in IL-1β production at 72 h (*p* = 0.05), while a strong trend to reduced production also remained at 4 weeks. This was not observed in the Pbo group (IL-1β, fold change from pre-infusion: 72 h, Pbo, 2.6 ± 1.8 versus IRL201805Res, 0.82 ± 0.6, *p* = 0.05; 4 weeks, Pbo, 1.52 ± 0.31 vs., 0.86 ± 0.56, *p* = 0.06 ns, n = 5) (Figure 6D); similarly, significantly lower IL-6 production at the 4 week time point was also detected (IL-6, fold change from pre-infusion: 72 h, Pbo, 2.5 ± 1.5 vs. IRL201805Res, 0.97 ± 0.5, *p* = ns; 4 weeks, Pbo, 1.28 ± 0.1 vs. IRL201805Res, 0.86 ± 0.35, *p* = 0.035, n = 5) (Figure 6E).

## 3. Discussion

The retrospective analysis of the RAGULA samples has highlighted several changes that clearly separated IRL201805Res patients from Pbo or IRL201805NRes, namely reduced serum IL-1β, TNFα, and IFNγ in parallel with increased sCTLA-4 indicative of regulation of the inflammatory response. Additionally, two constant changes in immune cell expression (i) CD39 on Tregs and (ii) increased intracellular IDO in monocytic cells were maintained over 12 weeks in IRL201805Res following IRL201805 infusion.

Despite the huge variability in endogenous serum BiP/GRP78 levels in health and disease, as in [4], it was noted that IRL201805Res tended to have higher levels than other patients. It was speculated that this increase in baseline endogenous BiP/GRP78 levels might aid the responsiveness of IRL201805Res patients by possibly enhancing self-antigen-recognising Tregs [16].

The production of serum cytokines is evidence of excessive cellular activation and an important indicator of the imbalance of the immune response that drives chronic inflammation. The successful use of anti-TNF therapy is evidence that this cytokine is a major driver of RA pathology. Having demonstrated that IRL201805 inhibits TNFα production and function, both in vitro [17] and in a xenogeneic severe combined immunodeficiency (SCID) mouse model with human synovial tissue transplants [18], the switch from pro- to anti-inflammatory mediators was targeted in this biomarker investigation. Overall, reduced serum IFNγ, already an established biomarker for successful anti-TNF therapy [19], and downregulated serum IL-1β and TNFα suggest an anti-inflammatory drift. When investigating the serum cytokine profile, one conundrum that arose was why the copious production of IL-10 was always observed in in vitro human PBMC cultures [17], and our primary in vitro biomarker before the clinical trial was not replicated in vivo. An explanation may be that, in vivo, secreted IL-10 is rapidly bound to receptors and consumed at the source. The significant decrease in serum IFNγ and TNFα, specifically in IRL201805Res patients, might suggest that IL-10 was active in the downregulation of nuclear factor kappa B (NF-κB), a transcription factor that drives production of many pro-inflammatory cytokines, and as expected would reduce pro-inflammatory cytokine transcription. This would correspond with the pre-clinical xenogeneic study where inflamed human synovial membrane was transplanted into SCID mice where the essential function of IL-10 was only confirmed using neutralizing IL-10 antibody [18].

Similarly, evidence of the deviation of monocyte development into alternatively activated macrophages with downregulation of antigen presentation by HLA-DR with costimulatory molecules CD86 and CD80 [20] also was previously observed in vitro [17] but was absent in the small group of IRL201805Res patients.

This shift away from a predominantly pro-inflammatory environment is accompanied by cellular changes. Monitoring CD3^+^CD4^+^CD25^hi^CD127^lo^ Treg [21], synonymous with Foxp3^+^positivity [22], showed no increase in Treg over 12 weeks in IRL201805Res patients. However, there was significant and prolonged upregulation of CD39 expression, known to increase Treg stability and potency. CD39, an ectoenzyme, regulates immune responses by hydrolysing ATP, increasing adenosine release which prevents T cell function and differentiation [23], ultimately making Treg function more effective and less reliant on Treg numbers. Low expression of CD39 is a biomarker for an inadequate response to methotrexate (MTX) [24]. Since several IRL201805Res patients had previously failed on MTX, but highly expressed CD39, these data strengthen the finding that IRL201805 has a different mechanism of action.

**Figure 7:**
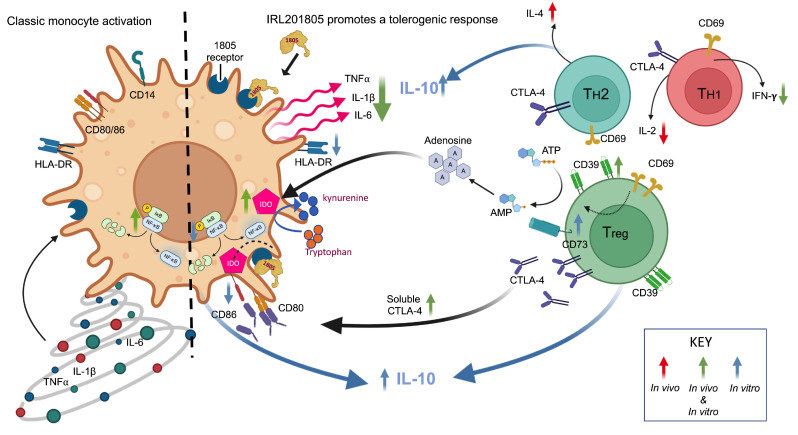
IRL201805 proposed mechanism of action promoting tolerogenic responses. A schematic cartoon of the proposed mechanism of action of IRL201805. **Left hand side** is of a classically activated monocyte expressing HLA-DR, CD86, and CD80 to present antigen to T cells and drive an inflammatory response with the production of IL-1β, IL-6, and TNFα. **Right hand side** is of the monocyte and shows the IRL201805-induced deactivation of the monocyte with reduced surface expression of HLA-DR and CD86. TNF, IL-1β, and IL-6 are downregulated. Although not found significantly in vivo, in vitro IL-10 is upregulated; IFNγ production is reduced. Increased release of sCTLA-4 blocks cell surface CD86 and CD80 required as costimulatory molecules in antigen presentation. sCTLA-4 causes the rapid induction of IDO. Decreased tryptophan inhibits T cell activation. Tregs are stabilized and have improved potency with expression of CD39 which digests ATP releasing adenosine which also downregulates T cell activity. Overall inflammatory cytokine release is reduced. The arrows show whether the biomarkers are increased or decreased, and the colour indicates whether the in vitro and ex vivo results are comparable or disparate.

Also, associated with Tregs, an anti-inflammatory mediator, soluble CTLA-4, was significantly increased in IRL201805Res following treatment for 12 weeks. This is relevant as CTLA-4 contains two single nucleotide polymorphisms associated with susceptibility to RA [25]. While CTLA-4 is essential for the maintenance of T cell subset homeostasis [26] and Treg activity, any genetic variation may alter this regulatory role in RA patients [26,27]. By increasing expression and secretion, IRL201805, may rebalance and restore immune regulation. Additionally, increased soluble CTLA-4 would mimic CTLA-4Ig function preventing T cell activation through costimulatory molecules, CD80 and CD86 [28]. Pre-clinical work has shown that IRL201805 downregulated CD86 sharply, and, although not observed in IRL201805Res patients, this may be due to variability of expression within the small patient numbers. CTLA-4 is also known to induce IDO, detected in monocytic cells [29] and which controls the degradation of tryptophan through the kynurenine pathway. Tryptophan is an essential amino acid for T cell proliferation [30] and metabolites also inhibit monocyte maturation to macrophages inducing a DC phenotype. The consequent upregulation of CTLA-4 and IDO and reduction in IFNγ would severely limit T cell activation and reduce inflammation (Figure 7).

IRL201805 is a synthetic homologue of human BiP, effectively a ‘self-antigen’, which as well as being a chaperone is a stress/heat shock protein (HSP) with evolutionary conserved features to bacterial HSPs recently reviewed [4]. Much of the pre-clinical work performed by Corrigall et. al, studying recombinant human BiP, observed changes in dendric cells characteristic of differentiation towards a tolerogenic DC (tolDC) phenotype [5,6,17,31,32,33]. Some of the features of tolDCs are low expression of CD40, CD80, CD86, and MHC class II molecules—all critical in the DC-T cell cross communication observed in both pro- and anti- inflammatory progression by promoting T cell anergy/depletion or Treg differentiation, respectively. TolDCs also generate potent inhibitory molecules including IDO and IL-10 among others. In this context, IRL201805 administration may work in part, by producing tolDC in vivo. Perhaps a more controlled way to generate tolDC is to manufacture them from autologous patients’ peripheral blood monocytes. The application of tolDC to restore immune tolerance has been tested in a series of completed phase I/II clinical trials in RA (NCT03337165/NCT01352858) [34], type 1 diabetes (NCT04590872), and multiple sclerosis patients (NCT02283671), and in most cases treatment was reported to be safe and well tolerated. The manufacture of autologous tolDC necessitates some ex vivo manipulation of the cells, including exposure to tolerogenic agents (e.g., Dexamethasone + Vitamin D3), a maturation agent to help cells display an anti-inflammatory profile [35] (e.g., monophosphoryl lipid A), and a specified antigen. An intra-nodal multi-dose 5–15 × 10^6^ tolDC (phase I/II) RA trial that is currently recruiting (NCT05251870) is employing a bacterial chaperone/HSP70 B-29 peptide at the antigen stimulant. This is of interest as the *Mycobacterium tuberculosis* B-29 peptide (VLRIVNEPTAAALAY) is believed to generate HSP-specific Tregs that suppress arthritis through the cross-recognition of human HSP homologues [36], and this is one aspect of IRL201805 we are aiming to assess in future studies to elucidate its mode of action further.

There are limitations of the biomarker analysis of this phase IIa randomised double-blind study. First, the samples size was limited to eight active RA patients per dosing arm (1, 5, or 15 mg single dose) who had all failed at least one DMARD. Of the 24 patients, 21 participants were women, potentially limiting the generalisability. There has been a rise in the placebo response in RA clinical trials over the past 20 years [37], with a possible explanation being RA severity has decreased over time due to early diagnosis and improved pharmacological intervention. As our patients were in a randomly selected double-blinded single-dose escalating study, our placebo group in general had higher DAS28 scores pre-infusion than our IRL201805 responders. Our Pbo group may reflect the undulating nature of the DAS28, which fluctuates despite the use of DMARDs. However, in the IRL201805Res, unlike the Pbo groups, they maintained a lower DAS28 score for the 12 week duration of the study.

The sum of the diverse biomarker changes observed upon treatment with IRL201805 in patients who responded to treatment indicated by reduced DAS28 scores, imply that not a single, but multiple immune and metabolic pathways may be altered by the drug in vivo. We are currently investigating this possibility by employing a number of molecular techniques to assess the in vitro action of IRL201805 on immune cells isolated from RA patients with active disease. We know that IRL1805 directly interacts with monocytes (see Figure 7) which are key cells that can initiate and regulate inflammation. In addition, monocytes can differentiate into osteoclast that are responsible for bone erosion in RA. Previously, we showed in healthy controls that IRL201805 inhibited osteoclastogenesis and its function by altering the mitogen-activated protein kinases (MAPKase) signalling pathway, by reducing phosphorylation of the p38 MAPKase, and the extracellular signal-regulated kinases (ERK) 1/2, with the suppression of essential osteoclast transcription factors, c-Fos and NFATc1, and finally preventing nuclear translocation of NF-κB components in both the canonical and non-canonical pathways [38]. We are currently performing in vitro RNA-seq analysis on monocytes from RA patients compared with healthy donors with and without IRL201805 treatment. So far, our evaluation of transcription profile changes in monocytes from active RA patients suggest that IRL201805 has the capacity to selectively reset a number of cell–cell and metabolic linked inflammatory pathways in RA monocytes, without altering the genes in healthy monocytes [39]. Moreover, upon further examination of osteoclasts derived from CD14+ monocytes isolated from active RA patients, in vitro application of IRL201805 appears to inhibit the receptor activator of nuclear factor-kB ligand (RANKL)-induced osteoclastogenesis and downregulate several vacuolar ATPases involved in the acidification of osteoclasts and bone resorption [40]. These observations support the biomarker changes observed in the former [38] and current study.

Biomarkers, common to both pre-clinical and clinical studies, indicate unequivocally that IRL201805 is an immunoregulator and that the long-term therapeutic properties of IRL201805, in the absence of a detectable drug, confirm a dissociation between the PK and PD. Figure 7 now places these exploratory biomarkers in context, explaining the multifaceted mechanism of action, and suggests that IRL201805 is a new class of biologics able to drive drug-free therapy by resetting the immune response and restoring homeostasis [4,41]. It appears that IRL201805 may target not one but multiple pathways common to many inflammatory and autoimmune diseases and joins the growing family of extracellular chaperone-derived molecules that may aid in the long-term remission of a number of chronic diseases and inflammation [42,43,44,45,46].

## 4. Materials and Methods

### 4.1. Reagents

IRL201805, an exact copy of clinical-grade GMP rhuBiP used in the RAGULA trial and a near homologue of endogenous BiP/GRP78, was re-manufactured by Revolo Biotherapeutics (Gaithersburg, MD, USA) and renamed IRL201805.

### 4.2. Study Design and Patient’s Characteristics

Twenty-four RA patients with active disease severity defined by their DAS28-ESR score who had failed one or more DMARDs were sequentially recruited to a double-blinded placebo-controlled, single-dose escalating phase I/IIA clinical trial for IRL201805 in RA. RA was defined using the 1987-revised ACR diagnostic criteria [47], for at least 6 months with active RA was defined by having at least six swollen and six tender joints, CRP  > 4 mg/L and/or ESR  > 15 mm/h, despite adequate dosage of at least one DMARD with a normal chest X-ray within 3 months of randomization. Major exclusion criteria were treatment with any biologic drug within 3 months of screening (6 months for rituximab), functional Class IV by ACR Criteria [48], safety screening pathology results outside pre-defined ranges, hepatitis B or C, HIV-positive status, and any other active systemic infection within 2 weeks before baseline. Patients with a history of malignancy (except basal cell carcinoma or adequately treated carcinoma in situ of the cervix), significant cardiac, renal, neurological, psychiatric, endocrine, metabolic, or hepatic disease were also excluded. Treatment regimes of the patients before and during the trial are reported in Table 1 of Ref. [7].

Six patients in each of the three dosing groups were infused with 1, 5, and 15 mg IRL201805/patient in a single dose. Patients were monitored for 12 weeks. In each group, two patients were randomly chosen to be placebo controls and were grouped at the end of the study. Due to the small number of patients, data were analysed in three groups across the dosing groups: (i) the placebo-treated group (Pbo), (ii) BiP-treated responder (IRL201805Res) patients, with a persistent moderate/good clinical response or in remission as measured using the DAS28-ESR from week 3 to week 12 and (iii) IRL201805-treated non-responder (IRL201805NRes) patients with no response to treatment but not withdrawn from the study due to worsening disease. Six patients were withdrawn during the trial, with four out of six for worsening disease, requiring treatment; two out of six withdrew due to spontaneous symptom relief. The variability of the pre-infusion DAS28.ESR scores between the three randomized groups was checked, employing the Kruskal–Wallis Test, and no significance was found (*p* = 0.0708). The median and IQR (Interquartile range) for each group were as follows: placebo, 5.2 (1.7); IRL201805Res, 4.4 (0.7); and IRL201805NRes, 6.0 (1.9). A pre-clinical in vitro study was also performed, in which heparinised blood samples from RA patients were collected from the Outpatients Clinic at Guy’s Hospital. All patients and healthy controls gave fully informed consent in line with the Helsinki Declaration and ethical standards for research. Research undertaken was approved by the Guy’s and St Thomas’ Hospital Research Ethics Committee (ref: 01/05/01).

### 4.3. Cell Culture

All cultures used tissue culture medium (TCM); (RPMI 1640) obtained from Sigma (Poole, UK) supplemented with 10% *v*/*v* foetal calf serum. PBMCs were prepared using Lymphoprep from Axis-Shield, (Dundee, UK), separated using density centrifugation, washed three times in phosphate-buffered saline (PBS) from BioWhittaker-Lonza (Cambridge, UK), and adjusted to 10^6^/mL/well in Corning Costar^®^ 24 well plates from Fisher Scientific (Loughborough, UK). When required, T cells were separated using negative selection using a Dynabeads™ untouched T cell purification kit from ThermoFisher Scientific (Waltham, MA, USA) according to manufacturer’s instructions. Heparinised blood samples from RA patients or healthy controls were obtained after fully informed consent from the rheumatology outpatient clinics at Guy’s Hospital and blood samples from the RAGULA trial were also processed for PBMCs (see details above). When required, PBMCs were frozen in 50% freezing medium (80% *v*/*v* foetal calf serum and 20% *v*/*v* dimethyl sulphoxide) and stored in liquid nitrogen.

### 4.4. Cytokines Production and Detection

PBMCs (10^6^/mL/well) were cultured in 24-well plates with and without stimulants, as indicated. Supernatants were collected, aliquoted, and frozen. An enzyme-linked immunosorbent assay (ELISA) kit used to detect IL-10 was obtained from Becton Dickenson (Oxford, UK). ELISA kits used for individual MMP3, TIMP1, sTNFR, and CTLA-4 detection were purchased from R&D Systems (Oxford, UK), and ELISA was performed according to each manufacturer’s recommendations. Serum levels of BiP were measured using ELISA, carried out as previously described [31]. A DuoSet^®^ commercial ELISA from R&D systems (Oxford, UK) was used to measure MMP-3/TIMP complexes. The high-variability cytokine concentrations in serum required normalisation of data for analysis. The fold change in cytokine concentration from the individual pre-infusion baseline concentration was used for each patient.

### 4.5. Cell Surface and Intracellular Flow Cytometry Analysis

Cell surface phenotypic analysis: in vitro PBMCs were stained for surface expression of CD45.fluorescein isothiocyanate (FITC), CD3 peridinin chlorophyll protein (Per-CP), CD4. phycoerythrin (PE), CD8.allophycocyanin (APC), CD69.PE, CD39.FITC, CD73.APC, CD14.PE, HLA-DR.FITC, CD86.FITC conjugated antibodies. For detection of CD39^+^ Treg ex vivo, whole blood was stained with a panel of fluorochrome conjugated antibodies from Biolegend^®^ (London, UK) including CD45.pacific blue, CD3.APC Cy7, CD4.FITC, CD25.APC, CD39.PE-Cy7, and live dead stain. CD127.PE was purchased from BD Biosciences (Oxford, UK). All antibody panels, clone numbers and fluorochrome compensation information are described in Table S1. After 30 min, red blood cells were lysed with isotonic ammonium chloride solution [48], and cells were washed three times in PBS and analysed within 1 h. Each sample was stained in parallel with an internal control to ensure technical consistency in the staining and analysis. The gating strategy employed to monitor phenotypic changes in CD39^+^ Tregs ex vivo is shown in Figure S3. Acquisition by BD CANTO II used application settings to ensure the consistency of results across the time points. Ex vivo monocytes and dendritic cells were cell surface stained for CD14, CD86, HLA-DR, CD1a, and intracellular IDO. All cells were stained as previously described [49].

Intracellular phenotypic analysis: in vitro PBMCs were cell surface stained for lymphocyte markers CD3.FITC and CD4.APC prior to being fixed with 4% paraformaldehyde and solubilised with 0.3% saponin, all purchased from Sigma (Poole, UK). Cells were counterstained with the appropriate fluorochrome conjugated antibodies to CTLA-4.PE or IDO.PE and washed thereafter in 0.3% saponin-containing buffer as previously described [33].

### 4.6. Ex Vivo Assessment of Patient Lymphocytes Retention of Antigen-Induced Proliferation and T-Cell Activation

Monitoring antigen-induced lymphocyte proliferation allows the assessment of the ability of immune cells to retain their ability to recognise ‘foreign’ antigens post treatment with tolerogenic therapy. PBMCs from all clinical time points were cultured in the presence of the recall antigen, tuberculin purified protein derivative (PPD) (5 µg/mL) from Central Veterinary Services (Weybridge, UK), or anti-CD3 and anti-CD28 antibody coated Dynabeads (20 beads/cell) from ThermoFisher Scientific (Paisley, UK). PBMCs (2 × 10^5^/200 µL/well) were set up in triplicate in Corning Costar^®^ 96-well plates (proliferation was measured using uptake of tritiated thymidine obtained from Perkin Elmer Inc., (Pierce, Beaconsfield, UK), as described previously) [32].

T cell activation via the TCR complex is required for the in vitro expansion of numerous immune signalling pathways. Treatment of T cells with monoclonal anti-CD3/anti-CD28 provides a generic co-stimulatory signal that engages the TCR. To investigate if lymphocytes post-treatment with IRL201805 in vivo retain their activation capabilities, RA trial PBMC secondary co-culture experiments were set up. Thawed autologous PBMC from the pre-infusion visit were mixed 1:1 with PBMCs (5 × 10^5^) from later time-points during the trial. All co-cultures for each patient were carried out simultaneously to ensure consistency. Cells were cultured for 72 h in the presence of a suboptimal dose of anti-CD3/CD28 antibody coated beads (20 cells:1 bead). Supernatants were aliquoted and frozen for cytokine detection.

### 4.7. Pharmacokinetic Determination

During the clinical trial, we were unable to measure PK in RA patients as blood collection, and both volume and frequency, were severely limited by the ethical committee. We knew from pre-clinical work that there was rapid uptake of IRL201805 (within a few hours) and an extended PD (several months); therefore, biomarker monitoring was a necessity to gauge drug efficacy. However, to confirm the rapid removal of IRL201805 from the circulation, it was relevant to measure the PK of the current drug batch in an animal model. Retrospectively, the PK study was performed by Aptuit (Verona, Italy) in rodents, as described below. In a GLP-compliant 7-day repeat-dose toxicity study, IRL201805 was administered to CD-1 mice at 0 (PBS vehicle), 5, 15, and 25 mg/kg/day by IV bolus injection for 7 consecutive days. The groups dosed for pharmacokinetic evaluation consisted of 9 mice/sex/group. Plasma samples were collected according to a composite profile of 3 mice/sex/timepoint on Day 1 and Day 7 at pre-dose, 1 and 10 min and 1, 5, 10, and 24 h after dosing. Plasma samples from animals given vehicle were collected at 1 min and 10 h after dosing.

Plasma concentrations of IRL201805 plus endogenous BiP were evaluated with a validated electrochemiluminescence assay (ECL) method suitable for the measurement of IRL201805 in mouse K2-EDTA plasma in the concentration range of 75.0–10,000 mg/mL.

Individual concentration data at the same nominal time were averaged where feasible and a composite concentration-time profile was constructed for each sex at each dose level. The following parameters were determined where feasible, using non-compartmental pharmacokinetic analysis, using the linear–logarithmic trapezoidal rule calculation method: C_max_, T_max_, t_1/2_, AUC_last_.

### 4.8. Statistical Analysis

SPSS Statistics for Windows (Version 22.0, IBM Corp, Armonk, NY, USA) or GraphPad Prism software version 10.2.2 were used for data analysis. Individual variables were assessed descriptively as median values and interquartile ranges. A non-parametric Mann Whitney test was used to assess the difference in median between the groups. To compare the medians for all three clinical groups a Kruskal–Wallis Test was used. To assess within-group comparison (between two time points), paired Wilcoxon or Spearman Rank tests were used. All *p*-values were two-sided and the significance level was set at 5% level. Statistics were supervised by Prof Toby Prevost Director of the Nightingale-Saunders Clinical Trials and Epidemiology Unit, a specialist section of the King’s Clinical Trials Unit (CTU).

## 5. Conclusions

IRL201805 joins the class of evolving biologic treatments that aim for the resolution of inflammation. IRL201805 has many modes of action, one of which is to enhance the generation of tolDC in vivo to treat RA and other autoimmune diseases. This is in contrast to conventional symptom-reducing immunosuppressive treatments. The development of therapeutics that have the capacity to reset immune responses specifically at targeted inflammatory sites, with minimal adverse side-effects, may be a future powerful and sustainable way to treat inflammatory and autoimmune diseases.

## Figures and Tables

**Table 1:** Comparative analysis of serum cytokine concentration in appropriate patients groups in the RAGULA clinical trial two weeks post-infusion of IRL201805.

Cytokine	Significance	Comparative Patient Groups
IFNγ	*p* = 0.02	Pbo>Res
TNFα	*p* = 0.085 (ns)	Pbo>Res
IFNγ	*p* = 0.03	NRes>Res
IL1β	*p* = 0.01	NRes>Res
TNFα	*p* = 0.054 (ns)	NRes>Res

**Pbo**, placebo; **Res**, IRL201805Res responders; **NRes**, IRL201805NRes non-responders. **Ns**, non significant.

## Data Availability

Data are contained within the article.

## Supplementary Materials

The following supporting information can be downloaded at https://www.mdpi.com/article/10.3390/ijms25084394/s1.
