# Supplementary material for: The Biologic IRL201805 Alters Immune Tolerance Leading to Prolonged Pharmacodynamics and Efficacy in Rheumatoid Arthritis Patients
Source: Int J Mol Sci. 2024 Apr 16;25(8):4394. doi: 10.3390/ijms25084394 (PMC11049849; doi:10.3390/ijms25084394)
Supplement: Supplementary file 1 [file ijms-25-04394-s001.zip › ijms-2927998-supplementary.pdf]

Supplementary Data.

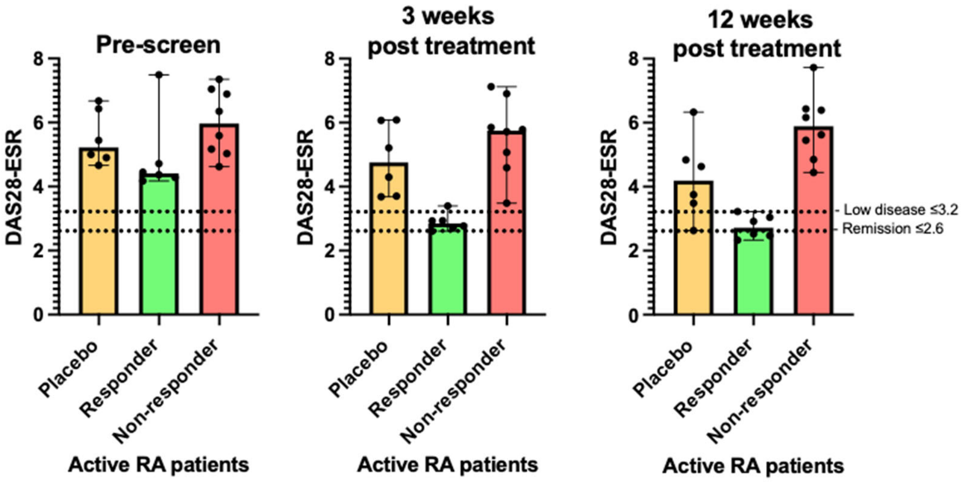

	Placebo	Responder	Non-responder	Placebo	Responder	Non-responder	Placebo	Responder	Non-responder
Median	5.2	4.4	6.0	4.8	2.9	5.8	4.2	2.7	5.9
IQR	1.7	1.1	1.9	2.4	0.4	1.9	1.9	0.7	1.4
Mann Whitney U	NS p= 0.09	NS p= 0.06		** p= 0.002	*** p= 0.0007		* p= 0.015	*** p= 0.0007	

Supplementary Figure S1. In the trial we used the DAS28 calculated with erythrocyte sedimentation rate (ESR) to assess disease activity. There was no statistically significant difference between randomised groups in the median DAS28.ESR ± IQR at the beginning of the trial. After three weeks onwards the IRL201805Res patients had a significantly lower DAS28 score compared to placebo and IRL201805NRes patients. At week 12 all responders (n =6) had achieved and retained a DAS28 score of between 3.2 and 2.6, defined as have low disease activity or remission respectively. In contrast, one placebo RA patient attained remission and no non-responders achieved low disease or remission status.

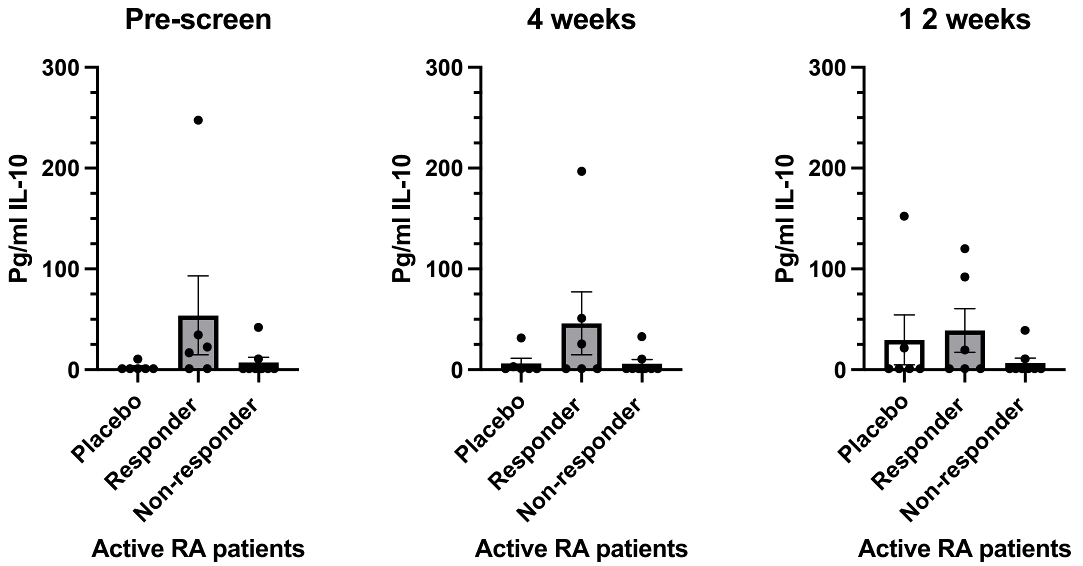

Supplementary Figure S2. IL-10 measurements in serum in RA patients at pre-screen, 4 weeks and 12 weeks after a single dose of either 1, 5 or 15 mg IRL201805 administered intravenously. Mean value ± SEM are reported.

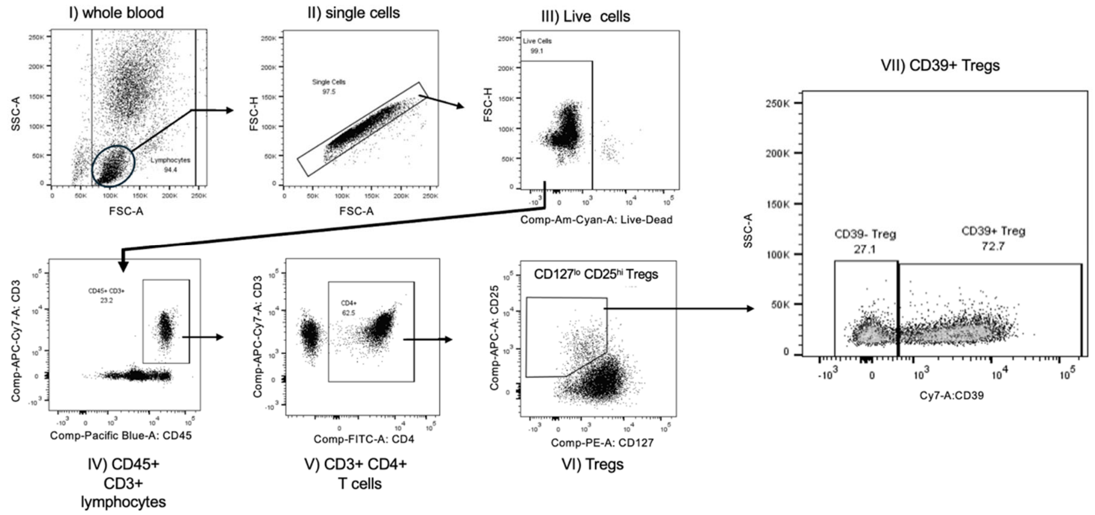

Supplementary Figure S3. Flow cytometry gating strategy for analysis of CD39+ Treg subsets. I) WBCs from whole blood were gated as, II) single cells and then III) viable cells were selected, IV) CD45+ lymphocytes were gated on CD45 and CD3. V) CD4+ lymphocytes were confirmed at the CD3+/CD4+ gate. VI) Tregs defined as CD25^{hi}CD127^{lo} were gated from the main population of T cells. VII) CD39+ cells were observed as a defined population at the SSC-A and CD39+ gate and compared between treatments.

Supplementary Table S1. Antibody panels used for flow cytometry staining

**Panel 1**	CD4/Treg			
Colour	Isotype	Antigen	*Cat. Number	clone
Pacific Blue	IgG1k	CD45	304021	HI30
APC Cy7	IgG1k	CD3	344817	SK7
FITC	IgG2bk	CD4	317407	OKT4
APC	IgG1k	CD25	302609	BC96
PE Cy7	IgG1k	CD39	328211	A1
PE	IgG1k	CD127	557938	hIL-7R-M21
**Panel 2**	CD8			
Pacific Blue	IgG1k	CD45	304021	HI30
APC Cy7	IgG1k	CD3	344817	SK7
FITC	IgG1k	CD8	344703	SK1
PE Cy7	IgG1k	CD28	302925	CD28.2
APC	IgG1k	CD56	318309	HCD56
PE	IgG1k	CD183	353705	G025H7
PerCP 5.5	IgG1k	CD62L	304823	DREG-56
**Panel 3**	Monocyte			
Pacific Blue	IgG1k	CD45	304021	HI30
PerCP 5.5	IgG1k	CD14	325621	HCD14
FITC	IgG2bk	CD20	302303	2H7
APC	IgG2bk	CD86	305411	IT2.2
PE Cy7	IgG2ak	HLA-DR	307615	L243
PE	IgG1k	CD80	305207	2D10

*All the panels: the antibodies were from Biolegend® except CD127 which is from BD Biosciences

Compensation of fluorochromes was performed using BD FACSDiva™ software on a BD FACSCanto™ which collected data from each compensation control tube and automatically calculates accurate compensation values for each fluorochrome combination. A typical compensation matrix for the Treg panel is shown below:

Show All	FITC-A	PE-A	PerCP-Cy5-5-A	PE-Cy7-A	APC-A	APC-Cy7-A	Pacific Blue-A	AmCyan-A
FITC-A	100	12.9232	3.1214	0.2552	0.2602	0.0773	0.4395	3.7702
PE-A	1.3602	100	17.06	0.7667	0.0197	0	0.1647	0.0726
PerCP-Cy5-5-A	0	0.039	100	9.468	3.2592	2.1753	0.1714	0.0838
PE-Cy7-A	0.4058	3.1773	8.9218	100	0.3647	7.7659	0.1756	0.1011
APC-A	0	0	3.954	0.2887	100	10.5857	0.1736	0.0992
APC-Cy7-A	4.2067	2.1406	2.1313	4.4673	9.5894	100	1.0006	0.5683
Pacific Blue-A	0.3743	0	0	0.0093	0.0334	0	100	30.8321
AmCyan-A	1.7286	0.3481	0.1833	0.0218	0	0	15.5819	100

The compensation controls were OneComp ebeads™ compensation beads (ebioscience, 01-1111-42) for the antibodies and patient cells for the fixable live dead control. Each drop of beads contains two populations: a positive population and a negative population that will

not react with species selected antibodies. Compensation controls were set up and run for each patient experiment.
